# A new diagnostic indication device of a biomarker growth differentiation factor 15 for mitochondrial diseases: From laboratory to automated inspection

**DOI:** 10.1002/jimd.12317

**Published:** 2020-10-04

**Authors:** Yasutoshi Koga, Nataliya Povalko, Eisuke Inoue, Akiko Ishii, Katsunori Fujii, Tatsuya Fujii, Kei Murayama, Yukiko Mogami, Ikue Hata, Masamichi Ikawa, Kei Fukami, Yoshihiro Fukumoto, Masatoshi Nomura, Kazuki Ichikawa, Kaori Yoshida

**Affiliations:** ^1^ Department of Pediatrics and Child Health Kurume University School of Medicine Kurume Japan; ^2^ Institute of Fundamental Medicine and Biology, Open Lab Gene and Cell Technology Kazan Federal University Kazan Russian Federation; ^3^ Showa University Research Administration Center, Showa University Tokyo Japan; ^4^ Department of Neurology Tsukuba University School of Medicine Tsukuba Japan; ^5^ Department of Pediatrics Chiba University Graduate School of Medicine Chiba Japan; ^6^ Department of Pediatrics Shiga Medical Center for Children Moriyama Japan; ^7^ Department of Metabolism Center for Medical Genetics, Chiba Children's Hospital Chiba Japan; ^8^ Department of Neurology Osaka Women's and Children's Hospital Izumi Japan; ^9^ Department of Pediatrics Fukui University School of Medicine Fukui Japan; ^10^ Department of Advanced Medicine for Community Healthcare, Faculty of Medical Sciences University of Fukui Fukui Japan; ^11^ Biomedical Imaging Research Center, University of Fukui Fukui Japan; ^12^ Division of Nephrology, Department of Medicine Kurume University School of Medicine Kurume Japan; ^13^ Division of Cardiovascular Medicine, Department of Internal Medicine Kurume University School of Medicine Kurume Japan; ^14^ Division of Endocrinology and Metabolism, Department of Internal Medicine Kurume University School of Medicine Kurume Japan; ^15^ Medical and Biological Laboratories Co., Ltd., Ina Laboratory Ina Japan

**Keywords:** biomarker, diagnostic indication device, GDF15, latex, LTIA, mitochondrial disease

## Abstract

Mitochondrial diseases (MDs) are occasionally difficult to diagnose. Growth differentiation factor 15 (GDF15) has been reported as a biomarker useful for not only diagnosing MDs, but also evaluating disease severity and therapeutic efficacy. To enable the measurement of serum GDF15 concentrations at medical institutions, we developed a new latex‐enhanced turbidimetric immunoassay (LTIA) as an automated diagnostic indication test for MDs. We also examined the equivalency of specificity and sensitivity in measuring serum GDF15 concentrations between a commercially available enzyme‐linked immunosorbent assay (ELISA) kit and a novel LTIA device in patients with MDs, disease controls, and healthy controls. A clinical performance study used a newly developed LTIA device and an existing ELISA kit to measure the concentrations of GDF15 in 35 MD patients, 111 disease controls, and 86 healthy controls. The median (first quartile‐third quartile) of serum GDF15 concentrations measured with the LTIA device was significantly higher (*P* < .001) in MD patients (1389.0 U/mL [869.5‐1776.0 U/mL]) than in healthy controls (380.5 U/mL [330.2‐471.8 U/mL]); the interquartile ranges did not overlap between MD patients and healthy controls. The areas under the curve in disease and healthy controls were 0.812 (95% confidence interval [CI]: 0.734‐0.886) and 0.951 (95% CI: 0.910‐0.992), respectively. The automated, high‐throughput technology‐based LTIA device has definite advantages over the ELISA kit in shorter processing time and lower estimated cost per sample measurement. The LTIA device of GDF15 may be a sufficiently reliable, frontline, diagnostic indicator of individuals with suspected MDs in the general population.

**Abbreviations**
BMI: body mass index
CK: creatine kinase
DM: diabetes mellitus
ELISA: enzyme‐linked immunosorbent assay
GDF15: growth differentiation factor 15
GFRAL: glial cell‐derived neurotrophic factor family receptor alpha‐like
HF: heart failure
KSS: Kearns‐Sayre syndrome
L/P ratio: lactate/pyruvate ratio
LS: Leigh syndrome
LTIA: latex‐enhanced turbidimetric immunoassay
MDs: mitochondrial diseases
MELA: mitochondrial encephalopathy and lactic acidosis
MELAS: mitochondrial encephalopathy, lactic acidosis, and stroke‐like episodes
RF: renal failure
ROC: receiver‐operating characteristic

**Synopsis**

A novel latex‐enhanced immunoturbidimetric assay device of GDF15 as the rapid frontline indication of MDs.

## 1 INTRODUCTION

Mitochondrial diseases (MDs) are occasionally difficult to diagnose because patients with MDs present with a constellation of symptoms (eg, psychomotor developmental delay, failure to thrive, short stature, convulsive seizures, muscle weakness, hearing loss, heart failure [HF], renal failure [RF], and diabetes mellitus [DM]).^1^ To date, several conventional biomarkers and markers (eg, lactate, pyruvate, creatine kinase, and lactate/pyruvate ratio) have been used to diagnose MDs in combination with clinicopathologic examinations and genetic testing. However, their sensitivity and specificity are not enough for making a definite diagnosis. To minimize a great number of individuals who are screened for MDs in clinical practice, therefore, the quick indication of MDs is a very important challenge for clinicians and diagnostic centers in terms of cost‐ and time‐efficiency before conducting genetic analysis.

Fibroblast growth factor 21 (FGF21) was identified as a biomarker of muscle‐manifested MDs in 2011.^2^, ^3^ Subsequently, growth differentiation factor 15 (GDF15) was discovered in thymidine kinase 2 deficiency patients^4^ and was confirmed as a biomarker by a number of studies.^5^, ^6^, ^7^, ^8^, ^9^, ^10^, ^11^, ^12^, ^13^, ^14^, ^15^ GDF15 is a member of the TGF‐β family, is expressed in numerous tissues, and seems to increase in response to stress signaling and inflammation.^16^ Furthermore, GDF15 was found to be increased in MDs' cybrids harboring the m.3243A>G, can be elevated in disorders other than MDs (eg, cardiovascular disorders), and is considered to be a good biomarker for MDs.^9^ The objective of the present clinical performance study was to examine the equivalency of specificity and sensitivity in measuring serum GDF15 concentrations between a commercially available enzyme‐linked immunosorbent assay (ELISA) kit and a novel latex‐enhanced turbidimetric immunoassay (LTIA) device used as a frontline indicator of individuals with suspected MDs in the general population.

## 2 PARTICIPANTS AND METHODS

### 2.1 Study design

The study protocol was approved by the institutional review boards of all medical institutions (coordinator: Kurume University, #16121). Written informed consent was obtained from all participants and/or their caregivers prior to enrolment. The study was conducted in accordance with the principles of the Declaration of Helsinki. This clinical performance study collected blood samples from participants between 2017 and 2020. All the samples were cryopreserved at −80°C until analysis.

### 2.2 Participants

Participants were categorized into either of the following groups: Group 1 (definitive MD patients) comprising individuals who met the clinicopathologic and genetic criteria for the diagnosis of MDs^17^ and Group 2 (asymptomatic individuals) who showed no symptoms but were found to have a known point mutation in the mitochondrial DNA. As disease controls, we recruited patients with neurodegenerative disorders who met the diagnostic criteria of the pertinent disorders, DM, HF, and RF. They were verified to have normal serum levels of lactate and pyruvate but did not undergo the genetic analysis of their mtDNA. We recruited Japanese healthy volunteers who had a normal body mass index as healthy controls.

### 2.3 LTIA device of GDF15

Two mouse monoclonal anti‐GDF15 antibodies developed by Medical & Biological Laboratories Co., Ltd. (MBL), Nagoya, Japan, were used in an in‐house sandwich ELISA and were compared with the R&D Systems GDF15 Quantikine ELISA.^18^ This verified that the MBL ELISA was comparable to the Quantikine ELISA (data not shown) and that the monoclonal antibodies were suitable for the LTIA device. The LTIA device of GDF15 is a newly developed tool for LTIA comprising latex microparticles (JSR, Tokyo, Japan) that are coated with these monoclonal antibodies. Serum GDF15 crosslinks latex microparticles via the coated antibodies, causing latex agglutination and a turbidity change. The calibration curve was obtained using the GDF15 calibrator and was used to calculate the values of the serum samples. To determine the kit calibration value, recombinant human GDF15 (957‐GD/CF; R&D systems, Minneapolis, MN) was used for comparison. GDF15 has no international standard. Therefore, the LTIA device calibrator's value was assigned by the R&D ELISA kit, corresponding to the cutoff value.^8^ These values were traceable to an in‐house standard.

### 2.4 GDF15 measurements

A manager of the study gave sequential numbers to the sera obtained from the collected blood samples, followed by blinded and randomized numbering prior to grouping. Subsequently, one clinical laboratory technician measured serum GDF15 concentrations in accordance with the previously reported method on the ELISA kit (Human GDF‐15 Quantikine ELISA Kit; R&D Systems).^8^ For the LTIA device, sample processing, pipetting, and measurements were performed with a fully automated laboratory analyzer, the BioMajesty JCA‐BM 8020 system (JEOL, Tokyo, Japan). Briefly, 10 μL of the sample and 60 μL of reagent 1 (reaction buffer solution) were injected into the reaction cuvette. After 5 minutes of incubation, reagent 2 (latex reagent) was added into the cuvette to start the immunoturbidimetric reaction. Absorbance changes in the reaction were measured at the wavelength of 805 nm from about 1 to 5 minutes after the addition of the latex reagent. Quantitation was performed based on the reaction rate. Serum GDF15 concentrations were measured with the LTIA device at MBL. Biospecimens, in which serum GDF15 concentrations exceeded the range of detection, were diluted tenfold with the dilution buffer before conducting the reanalysis.

### 2.5 Statistical analysis

The medians of serum GDF15 concentrations were calculated for the ELISA kit and the LTIA device. Between‐group comparisons were made according to the Mann‐Whitney's *U*‐test. The diagnostic indication test for MDs using the ELISA kit and the LTIA device was analyzed with the receiver‐operating characteristic (ROC) curve. The area under the curve (AUC) and its 95% confidence intervals (CIs) were estimated. The cut‐off value was determined using the Youden index method that derived the sensitivity and specificity of the ELISA kit and the LTIA device.^19^ Spearman's correlation coefficients were used to examine the associations between these measurement tools. A two‐tailed *P* value of <.05 was considered statistically significant. All statistical analyses were conducted with the SAS software package version 9.2 (SAS Institute, Cary, North Carolina).

## 3 RESULTS

### 3.1 Patients' demographic and clinical characteristics

**FIGURE 1:**
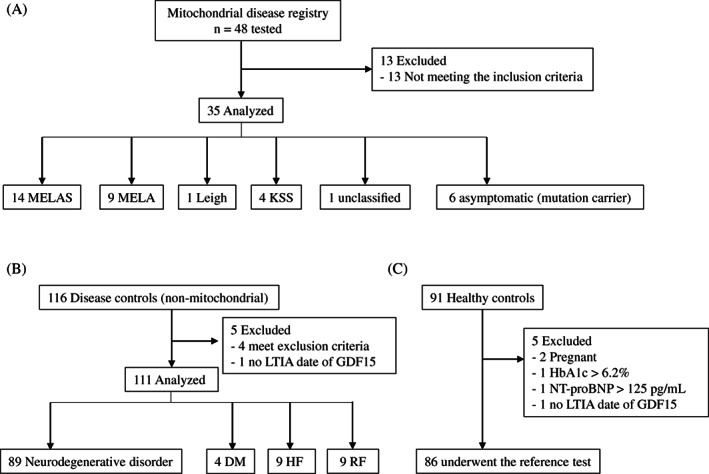
STARD flow diagram of patients with mitochondrial diseases, disease controls, and healthy controls. A, Forty‐eight patients with mitochondrial diseases, who met the clinicopathologic diagnostic criteria, were enrolled between 2017 and 2020. B, One hundred and sixteen disease controls who had nonmitochondrial diseases, 89, 4, 9, and 9 of whom had neurodegenerative diseases, DM, HF, and RF, respectively. C, Ninety‐one healthy controls were recruited, 86 of whom underwent the reference test. DM, diabetes mellitus; HF, heart failure; KSS, Kearns‐Sayre syndrome; LS, Leigh syndrome; MELA, mitochondrial encephalopathy and lactic acidosis; MELAS, mitochondrial encephalopathy, lactic acidosis, and stroke‐like episodes; and RF, renal failure

The blood samples collected from the following recruited individuals—48 MD patients, 116 disease controls, and 91 healthy controls—were tested with the ELISA kit and the LTIA device (Figure 1). Of 48 patients, 13 were excluded because of not meeting the clinical and genetic criteria. Finally, this study enrolled the following 35 patients: 14 and 9 who had MELAS and MELA, respectively—both of which are caused by either the m.3243A>G, m.13513T>C, or m.3250T>C in mtDNA, 1 had Leigh syndrome (LS) caused by the m.8993T>C mutation, 4 had Kearns‐Sayre Syndrome (KSS) due to the common 5‐kb single deletion, 1 had a defined respiratory chain enzyme deficiency, underwent genetic analysis that did not find any genetic alteration, and does not fit to any clinical type, and 6 were asymptomatic individuals who carried either a m.3243A>G mutation, a m.3303C>T mutation, or a m.3250T>C mutation in mtDNA (Figure 1A). One hundred and eleven disease controls consisted of adult and pediatric patients who did not have MDs. Concretely, 89 had neurodegenerative disorders including myasthenia gravis (n = 14), polymyositis (n = 7), multiple sclerosis (n = 6), Parkinson's disease (n = 5), Becker type muscular dystrophy (n = 2), limb‐girdle type muscular dystrophy (n = 3), and myotonic muscular dystrophy (n = 1). Furthermore, 4, 9, and 9 patients had DM, HF, and RF, respectively (Figure 1B). Patients with HF or RF were diagnosed based on an ejection fraction of <30%, and an estimated glomerular filtration rate of <60 mL/min/1.73 m^2^, respectively. Ninety‐one healthy controls, who did not receive any treatment or ambulatory medical care, were also recruited from MBL, Japan. However, four volunteers were excluded as follows: two had pregnancy, one had a hemoglobin A1c >6.2% who was diagnosed with DM, and one had an NT‐proBNP of >125 pg/mL (Figure 1C). MD patients, disease controls, and healthy controls are shown in Table 1. The medians (first quartile‐third quartile) of serum GDF15 concentrations, which were measured by ELISA and LTIA in healthy controls, MD patients, and disease controls, were obtained (Table 1). In MD patients, serum GDF15 concentrations were higher in patients with MELA, KSS, MELAS, and LS than in healthy controls. There were no significant differences in serum GDF15 concentrations among patients with MELA, KSS, or MELAS. Serum GDF15 concentrations did not differ between a patient with genetically unclassified MD—who showed elevated serum lactate concentrations and normal GDF15 concentrations—and healthy controls; the concentrations were significantly higher in asymptomatic individuals than in healthy controls. On the other hand, asymptomatic individuals who had normal plasma lactate concentrations showed slightly elevated serum GDF15 concentrations that were measured with the ELISA kit and the LTIA device. The medians of serum GDF15 concentrations measured with the ELISA kit and the LTIA device were higher (*P* < .001) in MD patients (Group 1) than in healthy controls. In disease controls, serum GDF15 concentrations were higher (*P* < .001) in patients with RF than in patients with neurodegenerative disorders, HF, or DM. On the other hand, serum GDF15 concentrations tended to be higher in patients with DM or HF than in healthy controls, but were significantly higher in patients with neurogenerative disorders than in healthy controls.

**TABLE 1:** Demographic, clinical, and laboratory characteristics of healthy controls, MD patients, and disease controls

		Disease categories	N	Age	Sex, (male %)	BMI, kg/m^2^	Serum GDF15 concentration		Lactate, mg/L	Pyruvate, mg/mL	L/P ratio	eGFR, mL/min per 1.73 m^2^	NT‐proBNP, pg/mL	HbA1C, %
							ELISA, pg/mL	LTIA, U/mL						
Healthy control		Total	86	33.0 [29.0‐40.0]	55.8	21.8 [19.6‐23.8]	247.2 [188.2‐303.1]	380.5 [330.2‐471.8]	9.0 [7.4‐11.6]	0.5 [0.4‐0.7]	16.15 [13.00‐21.09]	84.2 [76.9‐91.0]	20.5 [13.0‐37.0]	5.15 [5.00‐5.30]
MD patient	Group 1	MELAS	14	20.5 [13.8‐38.5]	57.1	16.6 [14.0‐17.8]	1731.7 [1233.5‐2278.6]	1482.0 [1226.2‐1711.0]	24.1 [21.2‐29.1]	1.2 [1.0‐1.4]	21.54 [18.64‐26.00]	128.7 [81.3‐204.3]	119.8 [86.8‐442.0]	4.95 [4.82‐6.22]
		MELA	9	54.0 [44.0‐63.0]	55.6	19.8 [17.7‐20.3]	2158.5 [1401.0‐3773.5]	1828.0 [1063.0‐2718.0]	18.4 [15.1‐30.2]	1.0 [0.8‐1.3]	19.73 [17.86‐23.97]	80.1 [77.8‐143.6]	59.0 [46.0‐249.0]	6.30 [5.10‐7.30]
		LS	1	3.0 [3.0‐3.0]	100	18.1 [18.1‐18.1]	1275.5 [1275.5‐1275.5]	1024.0 [1024.0‐1024.0]	14.5 [14.5‐14.5]	0.5 [0.5‐0.5]	29.00 [29.00‐29.00]	429.0 [429.0‐429.0]	698.0 [698.0‐698.0]	4.10 [4.10‐4.10]
		KSS	4	20.5 [18.0‐25.8]	50	19.7 [17.7‐21.2]	1726.8 [1467.4‐2126.5]	1541.5 [1362.5‐1819.2]	19.5 [16.7‐20.4]	0.9 [0.8‐0.9]	24.60 [20.38‐26.03]	144.1 [141.1‐147.2]	57.0 [46.5‐99.0]	5.30 [5.05‐5.40]
		Unclassified	1	9.0 [9.0‐9.0]	100	–	489.6 [489.6‐489.6]	381.0 [381.0‐381.0]	34.8 [34.8‐34.8]	2.4 [2.4‐2.4]	14.68 [14.68‐14.68]	266.8 [266.8‐266.8]	17.0 [17.0‐17.0]	5.50 [5.50‐5.50]
	Group 2	Asymptomatic	6	33.0 [21.5‐37.0]	16.7	17.2 [16.5‐18.2]	781.0 [617.2‐1073.1]	704.5 [528.8‐716.0]	11.9 [7.0‐14.4]	0.6 [0.5‐0.8]	16.27 [12.32‐22.19]	113.7 [107.8‐127.9]	86.5 [39.8‐146.0]	5.50 [5.17‐5.75]
		Total	35	34.0 [17.0‐47.5]	48.6	17.8 [15.8‐20.0]	1417.9 [1036.7‐2273.7]	1389.0 [869.5–1776.0]	21.1 [14.6‐29.1]	1.0 [0.8–1.3]	21.09 [16.57‐26.03]	121.0 [80.4‐153.8]	107.0 [49.2‐234.8]	5.25 [4.90‐6.30]
Disease control		Neuorodegenerative	89	54.0 [34.0‐68.0]	47.2	21.4 [17.1‐25.1]	517.6 [330.4‐819.8]	510.0 [341.5‐710.2]	12.5 [8.5‐17.7]	0.7 [0.5‐1.1]	16.35 [13.19‐20.04]	88.7 [62.9‐145.5]	62.2 [24.6‐124.5]	5.50 [5.30‐5.80]
		DM	4	43.5 [32.2‐53.5]	75	29.0 [25.8‐30.5]	579.5 [401.0‐1052.2]	482.5 [421.2‐709.8]	12.1 [10.9‐12.6]	1.3 [1.1‐1.3]	9.53 [9.42‐9.69]	92.1 [77.9‐112.4]	17.0 [11.2‐24.0]	8.40 [7.83‐9.23]
		HF	9	56.0 [46.0‐61.0]	77.8	22.7 [20.8‐25.8]	535.8 [425.9‐1019.0]	679.0 [433.0‐1143.0]	7.8 [6.5‐10.4]	0.7 [0.5‐1.0]	11.52 [10.16‐15.51]	67.7 [61.6‐79.3]	440.0 [233.0‐3910.0]	6.00 [5.40‐6.80]
		RF	9	55.0 [40.0‐71.0]	44.4	21.8 [17.1‐24.7]	4381.5 [1908.4‐5967.7]	2435.0 [1392.0‐3416.0]	12.2 [11.6‐13.2]	1.0 [0.9‐1.1]	11.01 [10.56‐12.06]	4.9 [4.0‐9.3]	2890.0 [1250.0‐4810.0]	6.10 [5.30‐7.00]
		Total	111	54.5 [36.0‐68.2]	50.9	22.1 [17.6‐25.5]	590.1 [346.7‐947.1]	548.0 [369.5‐788.5]	12.1 [8.2‐15.6]	0.8 [0.5–1.1]	14.94 [11.51‐18.93]	83.2 [61.4‐132.2]	83.0 [27.5‐300.8]	5.60 [5.30‐6.10]

*Note*: Values are expressed as median [Q1‐Q3].

Abbreviations: BMI, body mass index; DM, diabetes mellitus; ELISA, enzyme‐linked immunosorbent assay; HbA1c, hemoglobin A1c; HF, heart failure; KSS, Kearns‐Sayre syndrome; L/P ratio, lactate/pyruvate ratio; LS, Leigh syndrome; LTIA, latex‐enhanced immunoturbidimetric assay; MDs, mitochondrial disorders; MELA; mitochondrial encephalopathy and lactic acidosis; MELAS, mitochondrial encephalopathy, lactic acidosis, and stroke‐like episodes; NT‐proBNP, N‐terminal pro‐brain natriuretic protein; and RF, renal failure.

### 3.2 Correlations of serum GDF15 concentrations measured with the ELISA kit and the LTIA device

**FIGURE 2:**
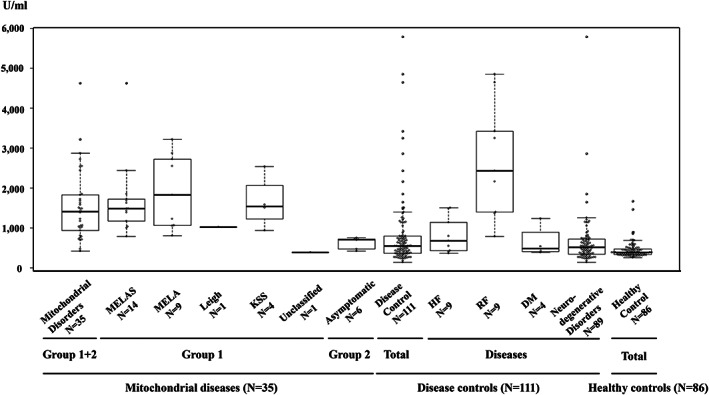
The box‐and‐whisker plots of serum GDF15 concentrations measured with the LTIA device in analyzed individuals. Patients with mitochondrial diseases included patients with MELAS, MELA, LS, or KSS, a patient with genetically unclassified MD, as well as asymptomatic individuals. Disease controls included patients with neurodegenerative disorders, DM, HF, or RF. HF, heart failure; KSS, Kearns‐Sayre syndrome; LS, Leigh syndrome; LTIA, latex‐enhanced turbidimetric immunoassay; MELA, mitochondrial encephalopathy and lactic acidosis; MELAS, mitochondrial encephalopathy, lactic acidosis, and stroke‐like episodes; RF: renal failure. The horizontal bold line, the box length, and the dotted line represent the median, the interquartile range, and the whiskers, respectively. The least and greatest values, as well as the outliers are also expressed. LTIA, latex‐enhanced turbidimetric immunoassay

**FIGURE 3:**
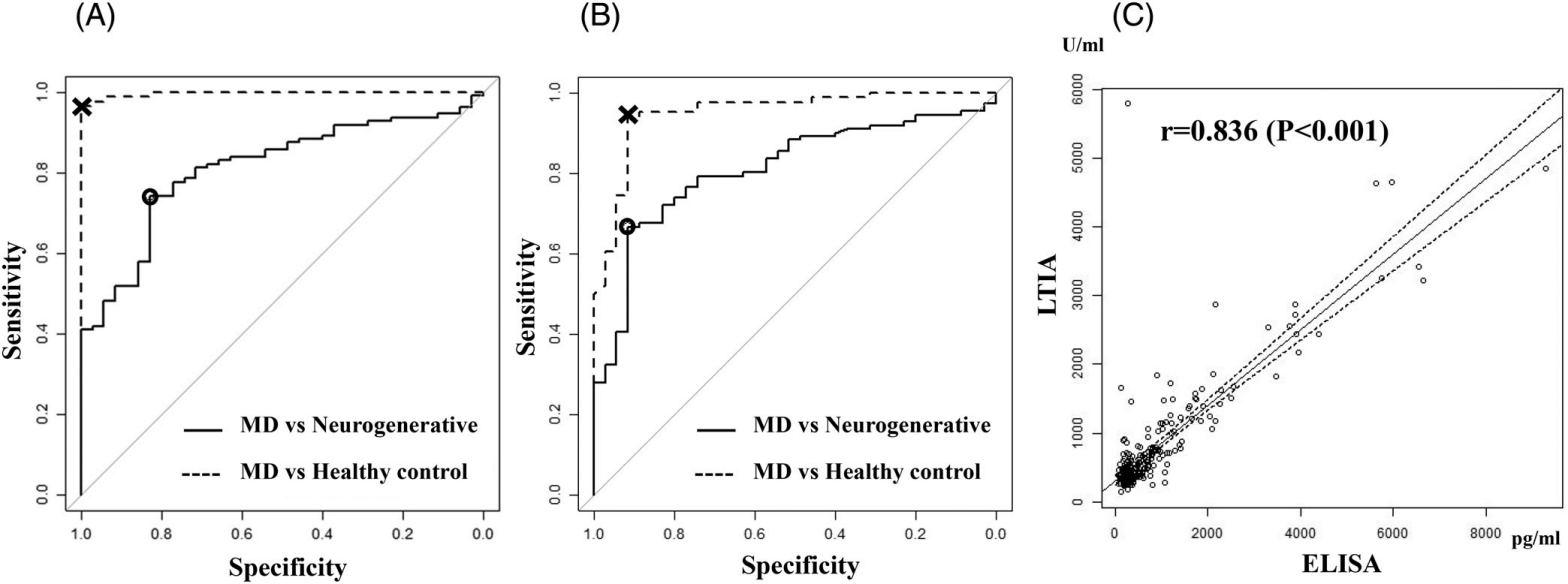
ROC analyses of serum GDF15 concentrations measured with the ELISA kit and the LTIA device in patients with MDs versus neurodegenerative controls and healthy controls. A, ROC curve of serum GDF15 concentrations measured with the ELISA kit. B, ROC curve of serum GDF15 concentrations measured with the LTIA device. C, Correlation of GDF15 concentrations between ELISA kit and the LTIA device. The solid line and dotted line in (A) and (B) show the ROC curve based on 35 patients with MDs versus neurodegenerative disorders and 35 patients with MDs versus healthy controls, respectively. Circles and crosses on (A) and (B) indicate points corresponding to cutoffs of Youden index based on the analysis of patients with MDs versus neurodegenerative controls and patients with MDs versus healthy controls, respectively. Youden index for patients with MDs versus neurodegenerative controls and patients with MDs versus healthy controls were 927.0 pg/mL and 476.0 pg/mL by ELISA, and 689.5 U/mL and 693.5 U/mL by LTIA, respectively. The solid line in (C) represents the correlation cure, and the dotted lines the 95% confidence interval. ELISA, enzyme‐linked immunosorbent assay; MD, mitochondrial disease; LTIA, latex‐enhanced turbidimetric immunoassay

The median differences in serum GDF15 concentrations measured with the ELISA kit and the LTIA device in MDs, neurodegenerative controls, and healthy controls are shown in Table 2. Serum GDF15 concentrations were significantly lower (*P* < .001) in Group 2 (asymptomatic individuals) than in Group 1 (definitive patients) and were significantly higher in Groups 1 and 2 than in the healthy control (*P* < .001). The box‐and‐whisker plots of GDF15 concentrations measured with the LTIA device in each group are shown in Figure 2. The interquartile ranges did not overlap between MDs and healthy controls. The results from the ROC analysis on serum GDF15 concentrations measured with the ELISA kit and the LTIA device in 35 patients with MDs, neurodegenerative controls, and healthy controls are shown in Figure 3A,B, respectively. The AUCs in healthy controls and neurodegenerative controls were as follows: by ELISA, 0.997 (95% CI: 0.992‐1.0) and 0.812 (95% CI: 0.739‐0.885), respectively; and by LTIA, 0.951 (95% CI: 0.910‐0.993) and 0.810 (95% CI: 0.734‐0.886). Youden index (cutoff values) for MDs versus neurodegenerative controls and MDs versus healthy controls were 927.0 pg/mL and 476.0 pg/mL by ELISA, and 689.5 U/mL and 693.5 U/mL by LTIA, respectively. The specificity and sensitivity for MDs when compared to healthy controls and neurodegenerative controls were as follows: by ELISA (Figure 3A), 1.00 and 0.97, 0.83, and 0.74, respectively; and by LTIA (Figure 3B), 0.91 and 0.94, 0.91, and 0.67, respectively. These results indicate the comparable diagnostic indication performance between the existing and novel devices for MDs. Serum GDF15 concentrations measured with the ELISA kit and the LTIA device were positively correlated (*r* = .836, *P* < .001; Figure 3C).

**TABLE 2:** Median differences between serum GDF15 concentrations measured with the ELISA kit and the LTIA device

Subject (n)	Category (n)	Median difference in serum GDF15 concentration				*P* value^a^ (ELISA vs LTIA)
		ELISA	*P* value (subject vs category)	LTIA	*P* value^a^ (subject vs category)	
Healthy control (86)	Mitochondrial disease (35)	−1170.6	<.001	−1008.5	<.001	.271
	Group 1 (29)	−1486.1	<.001	−1103.5	<.001	.246
	Group 2 (6)	−533.8	<.001	−324.0	.003	.394
	Neurodegenerative disease (89)	−270.3	<.001	−129.5	.006	.721
Mitochondrial disease (35)	Neurodegenerative disorder (89)	900.3	<.001	879.0	<.001	.794

*Note*: Group 1: definitive mitochondrial disease matched with clinical subtype; Group 2: asymptomatic individuals who have a pathogenic point mutation of mitochondrial DNA.

Abbreviations: ELISA, enzyme‐linked immunosorbent assay; GDF15, growth differentiation factor 15; LTIA, latex‐enhanced turbidimetric immunoassay.

a According to Mann‐Whitney *U* test.

## 4 DISCUSSION

### 4.1 Usefulness of GDF15

Several previous studies have reported the usefulness of GDF15 for the diagnostic indication of MDs. Koene et al. reported that serum GDF15 concentrations moderately correlate with MD severity and myocardial strain, but not with disease progression, in adult m.3243A>G carriers.^15^ Serum GDF15 concentrations are elevated in sorts of disorders—DM, cardiomyopathy, renal impairment, and cancer.^20^, ^21^, ^22^ Montero et al. reported that GDF15 is a sensitive and specific biomarker to guide the diagnosis of MDs in children.^14^ Serum GDF15 concentrations increased extensively in patients with MDs who had renal, cardiac, and/or CNS disorders concurrently. Normal serum GDF15 concentrations have been found in some patients with MDs. The reason for the finding is unclear and requires further investigation. However, the fact that the mutation load differs among patients' organs—which is translated into the clinical heterogeneity of MDs—may be a plausible causation. It would be of diagnostic indication help to measure the residual activity of the respiratory chain complex or the corresponding enzyme's activity in these patients. According to our data, elevated GDF15 concentrations were detected not only in the serum, but also in the cerebrospinal fluids of patients with MDs affecting the CNS (data not shown). This is a great diagnostic indication advantage of GDF15^8^ as compared with FGF21.^3^ MDs involving neurological presentation are also detectable by measuring serum GDF15 concentrations. Although the functional link between GDF15 and mitochondrial dysfunction remains to be definitely elucidated by future studies, we believe that serum GDF15 concentrations in patients with MDs may reflect mitochondrial dysfunction in various somatic cells of the human body.

Glial cell‐derived neurotrophic factor family receptor alpha‐like (GFRAL)—a distant relative of the receptors for a distinct class of the TGF‐β superfamily ligands that were found in 2017 to be located in the CNS of mice and to be most enriched in the hindbrain^23^—is very important for food intake control and body weight regulation in mice and humans.^24^ Blocking the interactions between GDF15 and GFRAL with a monoclonal antibody prevented the metabolic effects of GDF15 in rats.^25^ The existence of the GDF15 receptors in the brain may be one of the reasons why GDF15, but not FGF21, is elevated in the cerebrospinal fluid of patients with MDs. GDF15 is expected to be a candidate drug for the treatment of metabolic syndrome in the future.

GDF15 measured with the LTIA device for automated inspection is a biomarker very useful for diagnosing MDs because of the following rationales: first, the specificity and sensitivity of the LTIA device are good enough for the frontline indication of individuals with suspected MDs. This device does not require specialists or technicians because the assay can be run on a number of different high‐throughput clinical biochemistry analyzer platforms (eg, Hitachi Labospect and Beckman Coulter DxC series). Moreover, the estimated cost per sample measurement is <25 USD for the LTIA assay against >75 USD for the Quantikine ELISA. Namely, our novel LTIA device was demonstrated to be clinically useful for discerning asymptomatic patients from patients with definite MDs, and to be applicable to the diagnostic indication test before conducting genetic analysis in the diagnostic algorithm of MDs.^10^

The biomarker‐based discernment of patients with suspected MDs, who are covered by the national healthcare and/or a social health supporting system, from healthy individuals is critically important in the clinical settings. The measurement of serum GDF15 concentrations by means of the LTIA device is very useful to identify patients with a known genetic aberration and to assess MD severity as reported previously.^8^, ^11^ Of high clinical relevance is the diagnostic indication of individuals who meet the clinicopathologic, radiological, and/or biochemical criteria for the diagnosis of MDs even when genetic confirmation is pending. We will file for the regulatory approval to manufacture the LTIA device of GDF15 in Japan, with an indication for the diagnosis of MDs.

### 4.2 Strengths and limitations

The LTIA device has definite advantages over the ELISA kit because of (a) shorter sample processing time—10 minutes, (b) requiring only 10 μL of serum for a reaction, (c) better operability owing to high‐throughput technology by means of the fully automated analyzer, and (d) lower estimated cost per sample measurement: <25 USD. This study has several limitations. Since MDs are very heterogeneous in clinical presentation, a relatively small group size and a wide spectrum of clinical presentations in the group of MD patients resulted in the higher intragroup variations and in reduced statistical power. Nevertheless, the group size of patients with MDs—very rare conditions that caused functional limitations and could hinder them from participating in research—was larger than previous studies. This study neither included patients with MDs of nuclear DNA origin nor considered patients with MDs of a greater spectrum of etiologies who are encountered in the clinical settings. Therefore, further research will be necessary to determine the clinical performance of the LTIA assay.

## 5 CONCLUSION

The present clinical performance study revealed that LTIA is highly accurate in discerning patients with MDs from healthy individuals and patients with neurodegenerative disorders. LTIA, which was slightly inferior to ELISA in measurement performance, is of clinical relevance in affording the simpler, more economical, less burdensome, quicker, and more convenient measurement of serum GDF15 concentrations than ELISA. Therefore, the LTIA device may be used as a sufficiently reliable, frontline, diagnostic indicator of individuals with suspected MDs in the general population. We also anticipate further improvements in the accuracy of LTIA.

### AKNOWLEDGMENTS

The authors thank to Shuichi Yatsuga, MD, PhD, Miyuki Kitamura, MD, Miss China Ohinata, and Mrs. Kaori Murakami for the management of collecting specimens. The authors are grateful to Satoshi Sakima, MD, for the linguistical review of the manuscript. This research was supported by Grant #JP25461571 and #JP18K07892 (Yasutoshi Koga) from the Japan Society for the Promotion of Science, by grants #JP17ek0109088 and #JP19ek0109336 (Yasutoshi Koga) from the Japan Agency for Medical Research and Development (AMED).

## CONFLICT OF INTEREST

No potential conflict of interest relevant to this article were reported.

## ETHICS STATEMENT

Details of approval: The study protocol was approved by the institutional review boards of all medical institutions (coordinator: Kurume University, #16121). Written informed consent was obtained from all participants prior to enrolment.

Patient consent statement: All patients with MDs, disease controls, and healthy controls received adequate information on this study prior to the study onset. The study was conducted in accordance with the principles of the Declaration of Helsinki.

Patent: Yasutoshi Koga has a patent as “GDF15: a new diagnostic biomarker for mitochondrial diseases” which has been registered as application No. PCT/JP2015/050833 on January 14, 2015 and was certificated for patent # 6711966 by Japan Patent Office on June 2, 2020 issued. Patent rights are assigned to the Tokyo Metropolitan Institute of Gerontology and Kurume University. Licensee is MBL.
